# Arthropod communities of insular (São Miguel Island, Azores) and mainland (Portugal) coastal grasslands

**DOI:** 10.3897/BDJ.13.e144915

**Published:** 2025-02-24

**Authors:** Hugo Renato M.G. Calado, António O. Soares, Ruben Heleno, Paulo A. V. Borges

**Affiliations:** 1 University of the Azores, cE3c- Centre for Ecology, Evolution and Environmental Changes/Azorean Biodiversity Group, CHANGE – Global Change and Sustainability Institute, Faculty of Science and Technology, Rua da Mãe de Deus, 9500-321 Ponta Delgada, S. Miguel, Azores, Portugal, Ponta Delgada, Portugal University of the Azores, cE3c- Centre for Ecology, Evolution and Environmental Changes/Azorean Biodiversity Group, CHANGE – Global Change and Sustainability Institute, Faculty of Science and Technology, Rua da Mãe de Deus, 9500-321 Ponta Delgada, S. Miguel, Azores, Portugal Ponta Delgada Portugal; 2 Centre for Functional Ecology, Associate Laboratory TERRA, Department of Life Sciences, University of Coimbra, Calçada Martim de Freitas, 3000-456, Coimbra, Portugal Centre for Functional Ecology, Associate Laboratory TERRA, Department of Life Sciences, University of Coimbra, Calçada Martim de Freitas, 3000-456 Coimbra Portugal; 3 IUCN SSC Atlantic Islands Specialist Group, Angra do Heroísmo, Azores, Portugal IUCN SSC Atlantic Islands Specialist Group Angra do Heroísmo, Azores Portugal; 4 University of Azores, CE3C—Centre for Ecology, Evolution and Environmental Changes, Azorean Biodiversity Group, CHANGE —Global Change and Sustainability Institute, School of Agricultural and Environmental Sciences, Rua Capitão João d’Ávila, Pico da Urze, 9700-042, Angra do Heroísmo, Azores, Portugal University of Azores, CE3C—Centre for Ecology, Evolution and Environmental Changes, Azorean Biodiversity Group, CHANGE —Global Change and Sustainability Institute, School of Agricultural and Environmental Sciences, Rua Capitão João d’Ávila, Pico da Urze, 9700-042 Angra do Heroísmo, Azores Portugal; 5 IUCN SSC Monitoring Specialist Group, Angra do Heroísmo, Azores, Portugal IUCN SSC Monitoring Specialist Group Angra do Heroísmo, Azores Portugal

**Keywords:** arthropods, Azores, mainland, coastal grasslands, sweeping net, invasive species

## Abstract

**Background:**

The data presented here is part of a doctoral project aimed at characterising and comparing arthropod diversity across biotic communities in coastal ecosystems. The present work provides an inventory of the arthropods recorded in two coastal grasslands ecosystems: the Portugal mainland and the Azores. Sampling was conducted on São Miguel Island (Azores Archipelago) as well as in the Sesimbra and Sines regions (Setúbal District, mainland Portugal). Thirty-one plots were set and visited four times, in spring and summer of 2022.

**New information:**

The specimens collected were sorted and catalogued into a total of 534 arthropod species and morphospecies. In total, 67 species were common to both ecosystems. A total of 13,515 specimens were counted in the two coastal grasslands. We registered three new records for the Azores (in São Miguel Island), all being exotic: *Aritranisdirector* (Thumberg, 1822) (Hymenoptera, Ichneumonidae), *Draeculacephalabradleyi*, Van Duzee, 1915 (Hemiptera, Cicadellidae) and *Isodontia* sp. Patton, 1880 (Hymenoptera, Sphecidae). This publication demonstrates the importance of coastal grasslands as reservoirs for some potentially invasive arthropod species.

## Introduction

Grasslands are diverse and dynamic ecosystem that has been extensively studied due to their ecological significance. They are particularly important for supporting rich and varied arthropod communities (Jones and Donnelly 2004, Feher et al. 2021). Arthropods in this ecosystem provide diverse ecosystem services, such as nutrient cycling, carbon sequestration and pollination (amongst others) which are extremely important for humans (Peters et al. 2016). In turn, coastal grasslands are also home to many communities which are still poorly studied in their composition (but see Calado et al. (2024b)).

In island environments, monitoring is crucial due to their vulnerability to anthropogenic impacts (Borges et al. 2018, Delgado and Riera 2020, Boieiro et al. 2024). Studying the distribution and abaundance of arthropod communities in coastal grasslands, alongside consistent monitoring, provides valuable insights into the extent and nature of human-induced effects on these fragile ecosystems.

Grasslands across Europe and North America have suffered extensive degradation, leading to a substantial decline in the ecosystem services they provide and the loss of many associated species (Peters et al. 2016, Wick et al. 2016, Bardgett et al. 2021). The same happens in different parts of the world. For example, the rapid economic growth in China over the last 30 years resulted in massive construction activities that have altered landforms, vegetation and waterways, leading to surface runoff, soil erosion, sedimentation and land degradation (Dai et al. 2022).

At the same time, commerce and tourism can bring many invasive species of plants and animals that are being introduced in several ecosystems and lead to a loss of endemic organisms (Borges et al. 2022a, Boieiro et al. 2024). As an agricultural region, the Azores Archipelago depends on the vital roles played by various organisms, particularly arthropod communities, in supporting crop productivity. The decline or loss of arthropod communities could greatly affect crop productivity, with losses of ecosystem services that could lead to devastating consequences in food security (Calado et al. 2024b). Moreover, as these coastal communities host many exotic species, they could be reservoirs of potential agricultural pests or invasive species (Boieiro et al. 2024, Calado et al. 2024b).

Understanding how communities are distributed within a given ecosystem allows us to adopt better and more assertive solutions for the problems they face. In this way, comparative studies are important tools to provide information for conservation and restoration strategies that lead to the mitigation or reversal of the problems caused by anthropogenic actions over the years (Boieiro et al. 2024, Calado et al. 2024b).

## General description

### Purpose

The primary objective of this publication is to provide a comprehensive inventory of terrestrial arthropods sampled from grasslands across São Miguel Island (Azores Archipelago), Sesimbra and Sines regions (Setúbal District in Portugal mainland). The dataset includes detailed information on the abundance, diversity and composition of arthropod communities, collected through the project's monitoring surveys.

## Project description

### Title

Phenotypic Plasticity of Pest and Biological Control Agents: Contrasting Mainland and Insular Coastal Ecosystems

### Personnel

The project was conducted by Hugo Renato M.G.Calado and led by António O. Soares, Paulo A.V. Borges and Ruben Heleno.

Fieldwork: São Miguel Island: Hugo Renato M.G. Calado; Mainland: Hugo Renato M.G. Calado.

Taxonomists: Paulo A. V. Borges and Hugo Renato M.G. Calado.

Database management: Hugo Renato M.G. Calado and Paulo A. V. Borges.

Darwin Core databases: Hugo Renato M.G. Calado and Paulo A. V. Borges.

### Funding

H.R.M.G.C. was funded by the Regional FRCT Ph.D. Grant M3.1.a/F/012/2021: Phenotypic Plasticity of Pest and Biological Control Agents: Contrasting Mainland and Insular Coastal Ecosystems. A.O.S. and P.A.V.B. were also funded by the projects Pluriannual Funding FCT-UIDB/00329/2020-2024 - DOI 10.54499/UIDB/00329/2020 (Thematic Line 1 – integrated ecological assessment of environmental change on biodiversity), Azores DRCT Pluriannual Funding (M1.1.A/FUNC.UI&D/010/2021-2024) and PAVB by the project AZORESBIOPORTAL – PORBIOTA (ACORES-01-0145-FEDER-000072.

## Sampling methods

### Sampling description

An area of 2,500 m^2^ (0.25 ha) was defined for each plot to carry out the sampling programme. Plots were selected in both regions to have a similar general substrate (rocky), latitude and elevation. Thirteen plots on São Miguel Island and eighteen on Portugal's mainland, distributed across the Setúbal District (Sesimbra – 12 and Sines – 6), were visited four times between spring and summer of 2022. A total of 121 samples were collected (50 in São Miguel and 71 on Portugal’s mainland).

### Step description

**Arthropod sampling**

Sweeping nets were used to collect arthropods (which included spiders, true bugs, ants, beetles and other insects) on both coastal grasslands (in the Azores and on Portugal's mainland). In each plot, a random sweeping session was carried out using a nylon mosquito net 40 cm in diameter, 50 cm in length, with 0.25 mm mesh and an effort of 15 min. was spent (3 min. for sweeping and 12 min. for processing and labelling of the collected material). All collected specimens were transferred to tubes with 99.8% ethyl alcohol for later sorting and identification in the laboratory.

**Morphospecies identification**

In the laboratory, all arthropod specimens were sorted into morphospecies and stored in 2 ml Eppendorf tubes with 99.8% ethyl alcohol. For each morphospecies, at least one exemplar was selected and photographed, using a LEICA S9i stereomicroscope with LAS X 5.2.1.27831, to create a photographic database to facilitate taxonomic identification.

All morphospecies of the Azorean samples were identified by the senior author (Paulo A.V. Borges) to species level, when possible. All species collected in the Azores were categorised into three colonisation categories following the last checklist of Azorean arthropods (Borges et al. 2022b): endemic, native non-endemic and introduced. In some cases, the colonisation status was undetermined. A database for both events and occurrences was created following the Darwin Core criteria.

## Geographic coverage

### Description

**Figure 1.**
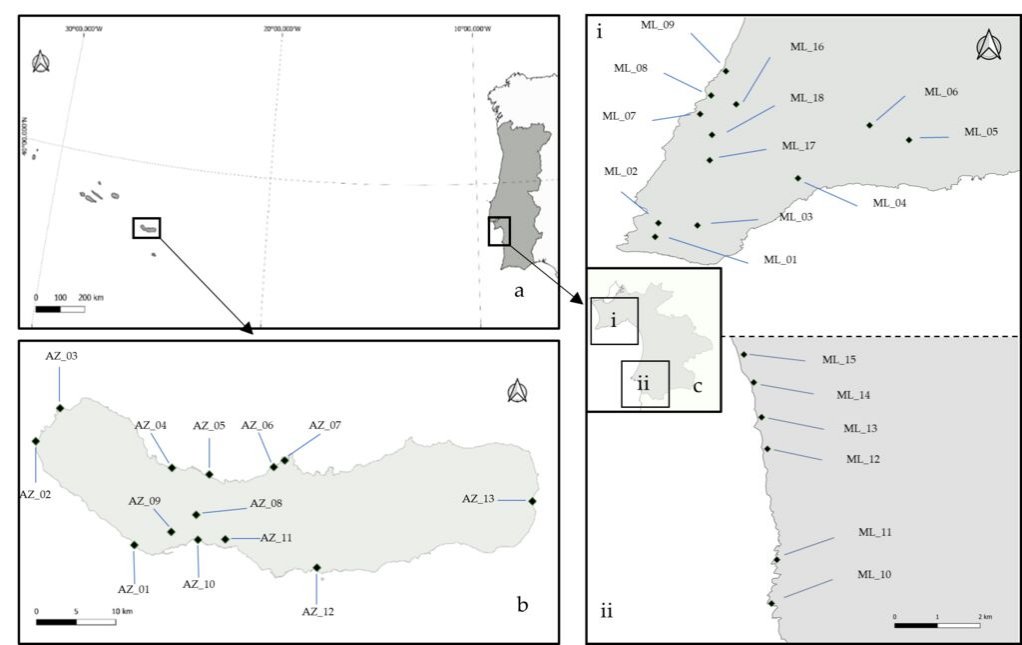
Sampling areas with the plots indicated: (**a**) Azores Archipelago and Portugal mainland; (**b**) São Miguel’s Island; (**c**) Setúbal District; (**i**) Sesimbra; (**ii**) Sines (source: Calado et al. (2024b)).

The study was conducted on the coastal grasslands of the São Miguel Inland (Azores Archipelago – North Atlantic) and Portugal Mainland (Setúbal District – Sesimbra and Sines regions) (Fig. 1).The Azores Archipelago is in the middle of the North Atlantic, approximately 1600 km from mainland Portugal, with an extension of about 600 km between Santa Maria and Corvo (37°–40° N latitude; 25°–31° W longitude). Three island groups compose the Archipelago: Eastern (Santa Maria and São Miguel Islands), Central (Terceira, Graciosa, São Jorge, Pico and Faial Islands) and Western (Corvo and Flores Islands). The different islands are aligned in a NW–SE orientation. São Miguel is situated in the oriental islands group (37.7804° N; -25.4970° W) and is the largest archipelago island with 746.8 km², measuring 64.7 km in length and 8–15 km in width and a maximum altitude above sea level of 1,103 m (Elias et al. 2016, Borges et al. 2019).

The Archipelago’s climate is affected by the surrounding ocean, namely, the effects of the Gulf Stream, as well as by island topography, being mild and very wet, often reaching an average annual relative humidity of 95% in high-altitude forests (Borges et al. 2019). The oceanic temperate climate is reflected in high annual precipitation, high relative humidity, persistent wind and low thermal amplitude (Pavão et al. 2023).

Mainland Portugal, on the other hand, is located in south-western Europe and is confined between parallels 37°N and 42°N and within the relatively narrow meridional band that develops between 6.5°W and 9.5°W. It lies in the transitional region between the sub-tropical anticyclone and the sub-polar depression zones. In this territory, the latitude, orography and effect of the Atlantic Ocean are the main driving forces of the climate (De Lima et al. 2015). The Setúbal District is located south of Lisbon, between parallels 37°N and 39°N. Sesimbra is ca. 30 km south of Lisbon and has an area of 194.98 km², whereas Sines is located ca. 150 km south of Lisbon and has an area of 195,47 km². Portugal has a Mediterranean climate characterised by warm and dry summers and cool and wet winters (Carvalho et al. 2014). Precipitation ranges from more than 2,000 mm in the northwest to roughly 400 mm in the most south-eastern part of the country (Santos et al. 2016).

## Taxonomic coverage

### Description

The following classes and orders are covered:

Arachnida: Araneae; Opiliones.

Diplopoda: Julida.

Insecta: Coleoptera; Diptera; Hemiptera; Hymenoptera; Lepidoptera; Mantodea; Neuroptera; Orthoptera; Phasmida; Psocodea; Thysanoptera.

## Temporal coverage

### Notes

The data were collected between March 2022 and 31 July 2022.

## Collection data

### Collection name

Renato_PhD

### Collection identifier

PHEPLA

### Specimen preservation method

99.8% ethyl alcohol

## Usage licence

### Usage licence

Creative Commons Public Domain Waiver (CC-Zero)

## Data resources

### Data package title

Arthropod communities of island vs. mainland coastal grasslands: São Miguel Island (Azores) and mainland Portugal.

### Resource link

http://ipt.gbif.pt/ipt/resource?r=matela_protest&v=1.1

### Number of data sets

2

### Data set 1.

#### Data set name

Event Table

#### Data format

Darwin Core Archive format

#### Character set

UTF-8

#### Download URL

http://ipt.gbif.pt/ipt/resource?r=matela_protest&v=1.1

#### Data format version

1.2

#### Description

The following data table includes all the records for which a taxonomic identification of the species was possible. The dataset submitted to GBIF is structured as a sample event dataset that has been published as a Darwin Core Archive (DwCA), which is a standardised format for sharing biodiversity data as a set of one or more data tables. The core data file contains 121 records (eventID). This GBIF IPT (Integrated Publishing Toolkit, Version 2.6.2) archives the data and, thus, serves as the data repository. The data and resource metadata are available for download in the Portuguese GBIF Portal IPT (Calado et al. 2024a).

**Data set 1.** 

Column label	Column description
eventID	Identifier of the events, unique for the dataset.
locationID	Identifier of the locations, unique for the dataset.
country	The name of the country or major administrative unit in which the Location occurs (Portugal).
countryCode	The standard code for the country in which the Location occurs (PT).
stateProvince	The name of the next smaller administrative region than country (state, province, canton, department, region etc.) in which the Location occurs.
county	The full, unabbreviated name of the next smaller administrative region than stateProvince (county, shire, department etc.) in which the Location occurs.
municipality	The full, unabbreviated name of the next smaller administrative region than county (city, municipality etc.) in which the Location occurs.
locality	The specific description of the place.
verbatimLocality	The original textual description of the place.
location Remarks	Comments or notes about the Location.
habitat	The habitat for an Event (coastal grasslands).
minimunElevationinMetres	The lower limit of the range of elevation (altitude, usually above sea level), in metres.
decimalLatitude	Approximate centre point decimal latitude of the field site in GPS coordinates.
decimalLongitude	Approximate centre point decimal longitude of the field site in GPS coordinates.
geodeticDatum	Standard Global Positioning System coordinate reference for the location of the sample collection points.
coordinateUncertaintyinMetres	Uncertain value of coordinate metrics.
coordinatePrecision	Value in decimal degrees to a precision of five decimal places.
georeferenceSources	Navigation system used to record the location of sample collections.
samplingProtocol	The sampling protocol used to capture the species (3 minutes of random sweeping at an area of 2,500 m^2^; 12 minutes to vacuum the organisms, put in flasks and label).
sampleSizeValue	A numeric value for a measurement of the size (time duration, length, area or volume) of a sample in a sampling Event.
sampleSizeUnits	The unit of measurement of the size (time duration, length, area or volume) of a sample in a sampling Event.
samplingEffort	The amount of effort expended during an Event (1 person randomly sweeping for 15 minutes on 0.25 ha plot, 4 repeats in spring and summer).
year	Year the sample was collected (2022).
month	The integer month in which the Event occurred.
day	The integer day of the month on which the Event occurred.
eventDate	The date-time or interval during which an Event occurred.
verbatimEventDate	The verbatim original representation of the date and time information for an Event.
dynamicProperties	Climatic conditions at the time of sampling at each location (Weather; Wind; AirTemperatureInCelsius; Nebulosity; Humidity).

### Data set 2.

#### Data set name

Occurrence Table

#### Data format

Darwin Core Archive format

#### Character set

UTF-8

#### Download URL

http://ipt.gbif.pt/ipt/resource?r=matela_protest&v=1.1

#### Data format version

1.2

#### Description

The dataset was published in the Global Biodiversity Information Facility platform, GBIF structured as an occurrence table that has been published as a Darwin Core Archive (DwCA), which is a standardised format for sharing biodiversity data as a set of one or more data tables. The core data file contains 3,636 records (occurrenceID). This GBIF IPT (Integrated Publishing Toolkit, Version 2.6.2) archives the data and, thus, serves as the data repository. The data and resource metadata are available for download in the Portuguese GBIF Portal IPT (Calado et al. 2024a).

**Data set 2.** 

Column label	Column description
eventID	Identifier of the events, unique for the dataset.
type	Type of the record, as defined by the Darwin Core Standard. In this case, "PhysicalObject".
licence	Reference to the licence under which the record is published.
institutionalID	The identity of the institution publishing the data.
collectionID	The identity of the collection publishing the data.
institutionCode	The code of the institution publishing the data (UAc).
collectionCode	The code of the collection where the specimens are conserved (PHEPLA).
datasetName	Name of the dataset (Renato_PhD).
basisOfRecord	The nature of the data record. In this case, "PreservedSpecimen".
recordedBy	A list (concatenated and separated) of names of people, groups or organisations who performed the sampling in the field.
occurenceID	Identifier of the record, coded as a global unique identifier.
datasetID	The identifier for the set of data.
organismQuantity	A number or enumeration value for the quantity of Organisms.
organismQuantityType	The type of quantification system used for the quantity of organisms.
kingdom	Kingdom name.
phylum	Phylum name.
class	Class name.
order	Order name.
family	Family name.
genus	Genus name.
specificEpithet	Specific epithet name.
infraspecificEpithet	Infraspecific epithet name.
scientificNameAuthorship	The authorship information for the scientificName formatted according to the conventions of the applicable nomenclaturalCode.
identificationRemarks	Comments or notes about the Identification (Morphospecie's number in Renato PhD Collection).
identifiedBy	A list of names of people, groups or organisations who assigned the Taxon to the subject.
dateIdentified	The date on which the subject was determined as representing the Taxon.
scientificName	The full scientific name, with authorship and date information if known.
taxonRank	Lowest taxonomic rank of the record.
establishmentMeans	The process of establishment of the species in the location, using a controlled vocabulary: 'native', 'introduced', 'endemic', "indeterminate".

## Additional information

**Figure 2.**
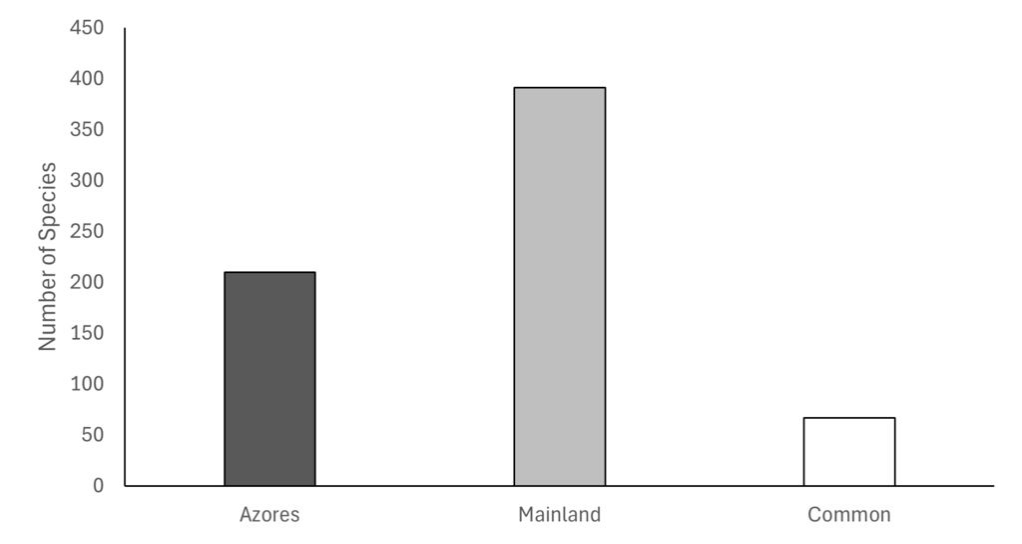
Number of species collected in the Azores and mainland coastal grasslands.

A total of 13,515 specimens were collected in the two coastal grasslands (Azores = 7861; Mainland = 5654) belonging to 534 arthropod species. In the Azores, 210 species were identified. Of those, 143 were found only in the Archipelago. For the mainland, 391 species were identified, with 324 present only there. A total of 67 species were common in both ecosystems (Fig. 2), (Table 1).

**Figure 3.**
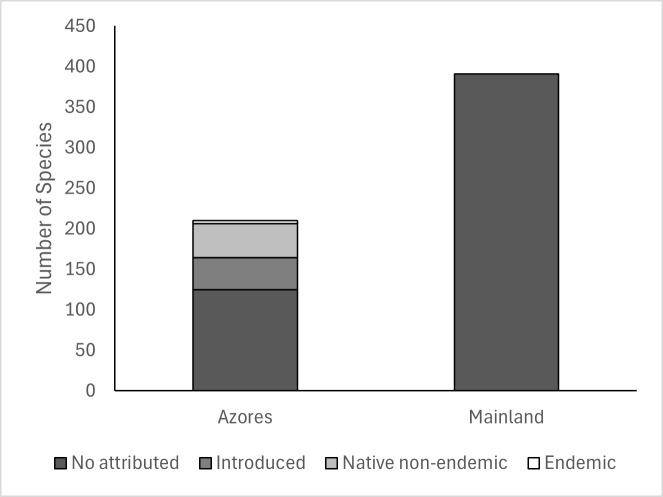
Total of species of terrestrial arthropods, organised by colonisation category, collected in the Azores and mainland coastal grasslands.

All the 210 taxa collected in the Azores were organised by colonisation category, following the last checklist of Azorean arthropods (Borges et al. 2022b). Of those, only four are endemic, with the others considered introduced (39), native non-endemic (42) or not yet specified (125) (Fig. 3).

Three new arthropod species were recorded for the Azores:

### Aritranisdirector (Thumberg, 1822) (Hymenoptera, Ichneumonidae)

The Ichneumonoidea is one of the largest superfamilies of the apocritan wasps with 58,121 described species and is distributed worldwide (Yu 2024).

This species is native to Europe and considered invasive in North America (Townes and Townes 1962). The length up to 10 mm, with a black head and thorax. The bulk of the abdomen is orange-red, with the final part black. The female, immediately before the ovipositor, also has a small white band at the tip of the abdomen. (Townes and Townes 1962). Females have a longer and downcurved ovipositor (longer than the metasoma) and the postpetiole is strongly convex (Verheyde et al. 2021).

**Figure 4.**
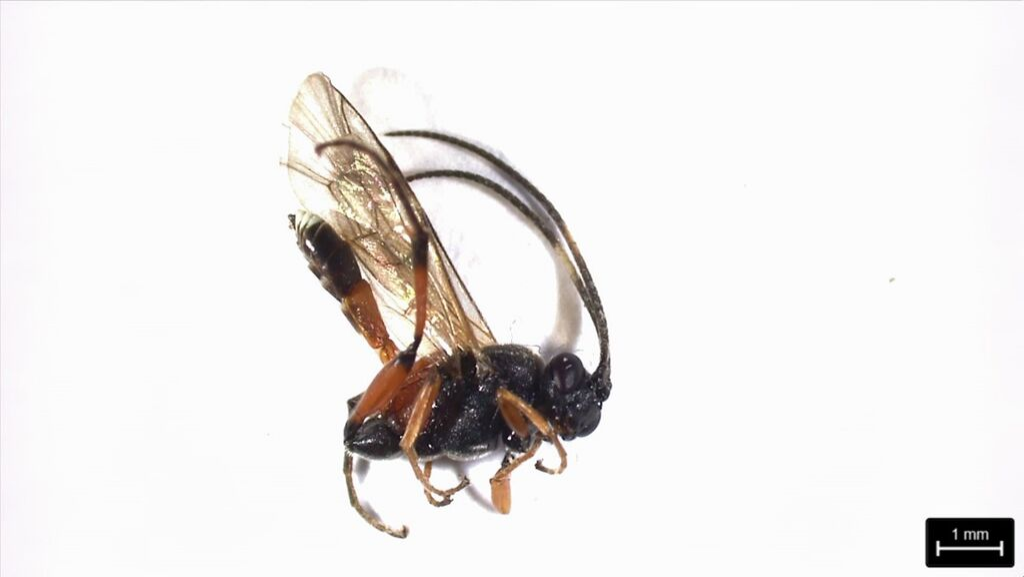
*Aritranisdirector* (Thumberg, 1822). Photo by Hugo Renato Calado.

This species was first found in grassland in São Roque (São Miguel Island), in April 2022, using a sweeping net (Fig. 4).

### Draeculacephala Ball (Hemiptera, Cicadellidae)

The genus naturally occurs throughout the temperate and tropical zones of North and South America, including some Caribbean islands, but some species have been introduced into Hawaii (Blanco-Rodríguez and Pinedo-Escatel 2022). *Draeculacephala* was established by Ball (1901) with Tettigonia mollipes as its type-species (Young 1977).

Draeculacephala can be easily recognised by its strongly depressed crown, which is typically angularly produced and its forewing, characterised by reticulated venation distally (Dietrich 1994, Blanco-Rodríguez and Pinedo-Escatel 2022).

**Figure 5.**
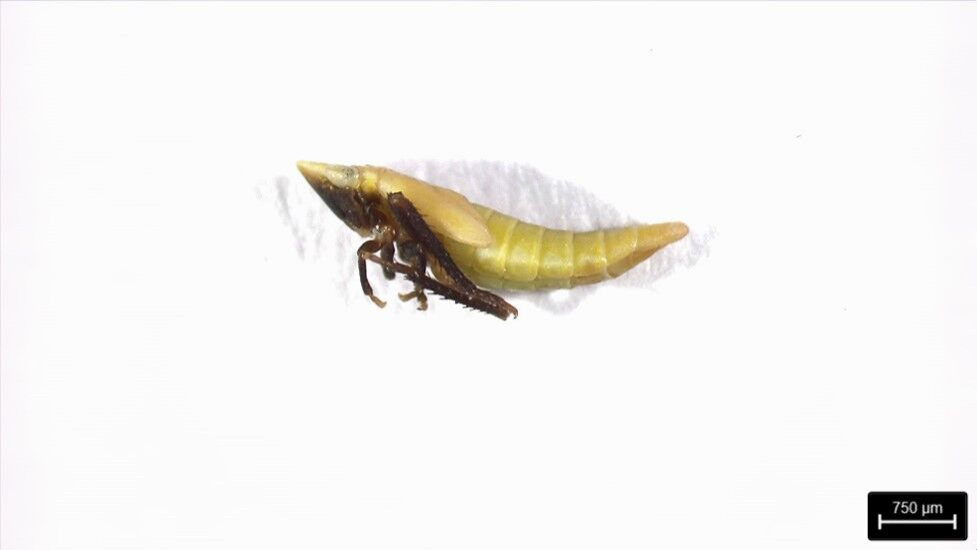
*Draeculacephalabradleyi*, Van Duzee, 1915. Photo by Hugo Renato Calado.

This species was first found in grassland in São Roque (São Miguel Island), in April 2022, using a sweeping net (Fig. 5).

### Isodontia sp. Patton, 1880 (Hymenoptera, Sphecidae)

**Figure 6.**
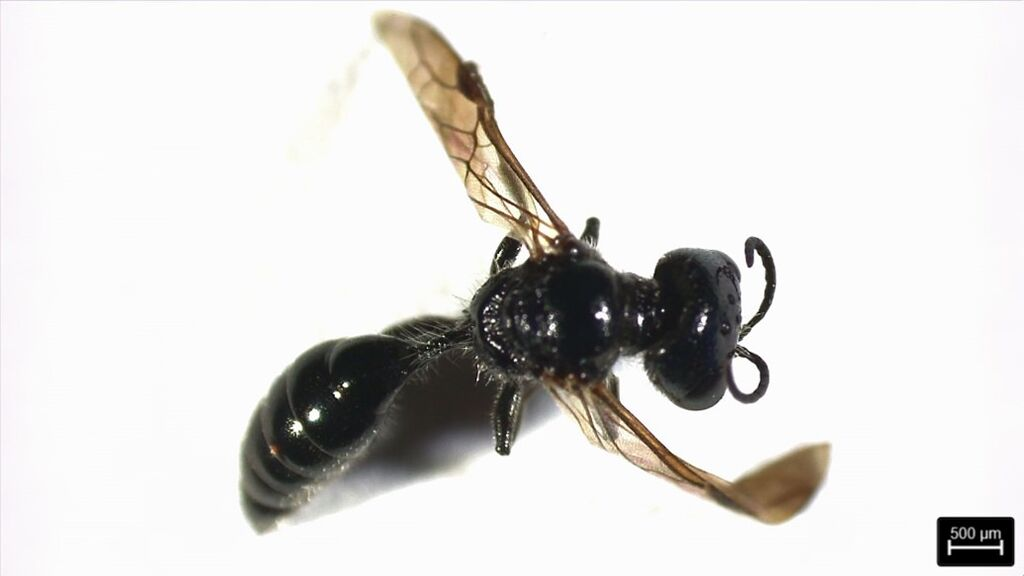
*Isodontia* sp. Patton, 1880. Photo by Hugo Renato Calado.

The genus *Isodontia* Patton, 1880 contains 62 described species distributed worldwide (Can 2024). The first recurrent vein enters the second submarginal cell, the second recurrent vein enters the third submarginal cell; the body is completely black (Notton 2017, Gladcaia 2024). This species was first found in grassland in São Roque (São Miguel Island), in April 2022, using a sweeping net (Fig. 6).

### Conclusions

This paper includes the inventory of the species collected in coastal grasslands in the Azores islands and Portugal's mainland in Calado et al. (2024b).

We observed greater arthropod diversity in the mainland coastal grasslands compared to those on São Miguel Island. The total number of species and morphospecies recorded in mainland coastal grasslands was significantly higher, in some cases doubling or exceeding those found in the Azores (e.g. for taxa such as Coleoptera, Hymenoptera and Araneae). These findings align with the well-documented pattern that insular ecosystems tend to be species-poor and exhibit disharmonic species composition (Whittaker et al. 2017).

Comparing different types of ecosystems provides valuable insights into species composition within communities, their distribution patterns and their ecological roles. This understanding is crucial for assessing the complexity of a given ecosystem and evaluating its potential vulnerability to anthropogenic impacts. Additionally, studies like this contribute to the broader understanding of local biodiversity, particularly for taxonomic groups that remain understudied, highlighting the need for further research to fill knowledge gaps (Borges et al. 2022b).

Therefore, through these studies, it will be possible to predict which species can be marked as potential invaders and the risks they may pose to native species. This information can be useful to decide the best preventative measures to impede their spread and mitigate potentialy harmful effects. At the same time, this will be helpful for minimising the costs related to eventual pest outbreaks (Nentwing 2008, Boieiro et al. 2024).

Finally, given the climate changes we are currently experiencing, long-term monitoring of these environments will also allow us to adopt the most effective measures to safeguard some species that are most sensitive to these same changes, as well as trying to predict which will be the results of the losses of their respective habitats.

## Figures and Tables

**Table 1.** Common species in the Azores and mainland coastal grasslands.

**Class**	**Order**	**Family**	**Species**	**Total Abundance Azores**	**Total Abundance Mainland**
**Arachnida**	Araneae	Araneidae	*Mangoraacalypha* (Walckenaer, 1802)	16	117
			*Neosconacrucifera* (Lucas, 1838)	8	1
			*Zygiellax-notata* (Clerck, 1757)	42	66
		Linyphiidae	*Oedothoraxfuscus* (Blackwall, 1834)	11	4
			*Prinerigonevagans* (Audouin, 1826)	52	15
		Salticidae	*Chalcoscirtusinfimus* (Simon, 1868)	73	13
			*Macaroerisdiligens* (Blackwall, 1867)	16	6
			*Salticusmutabilis* Lucas, 1846	31	28
			*Synagelesvenator* (Lucas, 1836)	61	7
		Thomisidae	*Xysticusnubilus* Simon, 1875	90	97
**Insecta**	Coleoptera	Apionidae	*Aspidapionradiolus* (Marsham, 1802)	72	3
		Chrysomelidae	*Psylliodesmarcida* (Illiger, 1807)	11	49
		Coccinellidae	*Rhyzobiuslitura* (Fabricius, 1787)	38	5
			*Scymnusinterruptus* (Goeze, 1777)	129	2
			*Scymnussuturalis* Thunberg, 1795	2	1
		Curculionidae	*Mecinuspascuorum* (Gyllenhal, 1813)	286	18
		Nitidulidae	*Brassicogethesaeneus* (Fabricius, 1775)	54	8
		Phalacridae	*Stilbustestaceus* (Panzer, 1797)	14	10
		Staphylinidae	*Tachyporuschrysomelinus* (Linnaeus, 1758)	1	1
	Diptera	Agromyzidae	*Chromatomyianigra* (Meigen, 1830)	10	1
		Calliphoridae	*Luciliasericata* (Meigen, 1826)	16	3
		Chloropidae	*Thaumatomyianotata* (Meigen, 1830)	32	35
		Lonchopteridae	*Lonchopterabifurcata* (Fallén, 1810)	67	69
		Muscidae	*Coenosiahumilis* Meigen, 1826	65	8
			*Muscaosiris* Wiedemann, 1830	70	6
			*Stomoxyscalcitrans* (Linnaeus, 1758)	123	71
		Opomyzidae	*Geomyzatripunctata* (Fallén, 1823)	4	3
		Rhinophoridae	*Melanophoraroralis* (Linnaeus, 1758)	82	15
		Syrphidae	*Eristalistenax* (Linnaeus, 1758)	4	3
			*Eupeodescorollae* (Fabricius, 1794)	15	7
			*Sphaerophoriascripta* (Linnaeus, 1758)	34	12
		Tephritidae	*Dioxynasororcula* (Wiedemann, 1830)	220	1
	Hemiptera	Anthocoridae	*Oriuslaevigatuslaevigatus* (Fieber, 1860)	44	3
		Aphididae	*Aphisfabae* Scopoli, 1763	58	2
			*Aphisnerii* Boyer de Fonscolombe, 1841	42	2
			*Melanaphisdonacis* (Passerini, 1862)	257	48
			*Myzuspersicae* (Sulzer, 1776)	127	45
			*Therioaphistrifolii* (Monell, 1882)	19	10
		Aphrophoridae	*Philaenusspumarius* (Linnaeus, 1758)	285	241
		Cicadellidae	*Macrostelessexnotatus* (Fallen, 1806)	301	14
		Delphacidae	*Megamelodesquadrimaculatus* (Signoret, 1865)	44	3
			*Sogatellanigeriensis* (Muir, 1920)	252	45
		Lygaeidae	*Kleidocerysericae* (Horváth, 1909)	49	6
			*Nysiusericaeericae* (Blackwall, 1867)	14	15
		Miridae	*Taylorilygusapicalis* (Fieber, 1861)	433	26
		Nabidae	*Nabiscapsiformis* Germar, 1838	180	3
		Pentatomidae	*Nezaraviridula* (Linnaeus, 1758)	53	13
		Psyllidae	*Acizziauncatoides* (Ferris & Klyver, 1932)	15	21
		Rhyparochromidae	*Beosusmaritimus* (Scopoli, 1763)	1	9
		Saldidae	*Saldulapalustris* (Douglas, 1874)	4	1
	Hymenoptera	Aphelinidae	*Encarsiaformosa* Gahan, 1924	9	2
		Apidae	*Apismellifera* Linnaeus, 1758	19	6
			*Bombusterrestris* (Linnaeus, 1758)	16	3
		Encyrtidae	*Pseudaphycusmaculipennis* Mercet, 1923	13	2
		Eulophidae	*Baryscapusgalactopus* (Ratzeburg, 1844)	69	86
			*Diglyphusisaea* (Walker, 1838)	6	4
		Formicidae	*Hypoponeraeduardi* (Forel, 1894)	5	5
			*Lasiusgrandis* Forel, 1909	593	6
			*Tetramoriumcaespitum* (Linnaeus, 1758)	20	4
		Ichneumonidae	*Aritranisdirector* (Thumberg, 1822)	1	1
			*Diplazonlaetatorius* (Fabricius, 1781)	32	4
		Mymaridae	*Lituscynipseus* Haliday, 1833	6	2
		Pteromalidae	*Pteromaluspuparum* (Linnaeus, 1758)	8	7
	Lepidoptera	Pieridae	*Coliascroceus* (Fourcroy, 1785)	4	1
	Orthoptera	Acrididae	*Locustamigratoria* (Linnaeus, 1758)	3	1
		Trigonidiidae	*Trigonidiumcicindeloides* Rambur, 1838	69	3
	Psocodea	Caeciliusidae	*Valenzuelaflavidus* (Stephens, 1836)	48	3
**Total**				**4848**	**1332**
